# Metasurface-Enabled 3-in-1 Microscopy

**DOI:** 10.1021/acsphotonics.2c01971

**Published:** 2023-01-26

**Authors:** Yuttana Intaravanne, Muhammad Afnan Ansari, Hammad Ahmed, Narina Bileckaja, Huabing Yin, Xianzhong Chen

**Affiliations:** † Institute of Photonics and Quantum Sciences, School of Engineering and Physical Sciences, Heriot-Watt University, EdinburghEH14 4AS, U.K.; ‡ National Electronics and Computer Technology Center, National Science and Technology Development Agency, 112 Thailand Science Park, Phahonyothin Road, Khlong Nueng, Khlong Luang, Pathum Thani12120, Thailand; § Biomedical Engineering Division, James Watt School of Engineering, University of Glasgow, GlasgowG12 8QQ, U.K.

**Keywords:** optical metasurfaces, edge imaging, polarimetric imaging, multifunctional microscopy

## Abstract

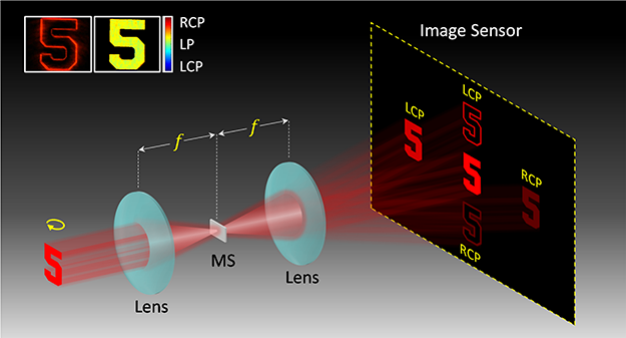

Edge enhancement and polarization detection are critical to image transparent or low-contrast samples. However, currently available systems are limited to performing only a single functionality. To meet the requirement of system integration, there is a pressing need for a microscope with multiple functionalities. Here, we propose and develop a microscope with three different functionalities based on spatial multiplexing and polarization splitting. A novel geometric metasurface (MS) is used to realize a spiral phase profile and two phase gradient profiles along two vertical directions, which can perform such an extremely challenging optical task. This is the first demonstration of a 3-in-1 microscope that can simultaneously obtain five images with different optical properties in an imaging plane for the same sample. Imaging experiments with different samples verify its capability to simultaneously perform edge imaging, polarimetric imaging, and conventional microscope imaging. Benefiting from the compactness and multifunctionality of the optical MS device, the integration does not increase the volume of the microscope. This approach can enable users to visualize the multiple facets of samples in real-time.

## Introduction

To decrease the light scattering from different tissue structures, biological samples used for microscopy imaging are typically very thin. These tissue samples are usually transparent, leading to little contrast under a microscope without image enhancement techniques.^1−3^ Distinguishing the edges of transparent biological samples precisely and enhancing the target detection and recognition performance of an imaging system are especially important.^4−6^ Although chemical staining is a very popular approach, these dyes can interact with samples in a way that obscures observation, either by altering or killing the samples. Since living systems use almost exclusively l-amino acids and d-sugars, essentially, most biomolecules (e.g., peptides, DNA, and collagens) consist of chiral structures,^7^ meaning that they are sensitive to circularly polarized light. Edge enhancement^8−10^ and polarization detection^11−15^ are critical to image transparent or low-contrast biological samples. However, currently available systems are limited to performing only a single functionality. In addition, imaging systems that combine multiple functionalities tend to be expensive and bulky because of the substantial footprint of their benchtop-based electronic and optical components, which reduce the portability and hinder their practical applications.^16^ Moreover, such imaging systems are often custom-fabricated and commercially unavailable. This makes them unsuitable for mass production or widespread adoption by biologists. There is a crucial need to develop portable microscopy systems that can simultaneously possess multiple functionalities.

Current edge imaging and circular polarimetric imaging suffer from technical and practical challenges. Phase contrast microscopy works by recombining and interfering with the directly transmitted and scattered light. Unlike typical phase contrast imaging, edge imaging is based on the spiral phase contrast technique, where the opposite halves of any radial line of the spiral phase element can introduce a phase difference of π between the positive and negative spatial frequencies of incident light field, leading to a strong isotropic edge contrast enhancement of observed amplitude and phase objects. Since it only extracts important information and records basic geometric features related to the edges of an object, the spiral phase contrast imaging greatly reduces the amount of data to be processed. To date, the primary realization of spiral phase contrast imaging is accomplished using liquid crystal-based spatial light modulators,^17−19^ which suffer from large volume, limited resolution, and high cost. On the other hand, most biomolecules have different responses to circularly polarized light with left and right handedness due to their chiral structures. The interaction between a chiral biomolecule and polarized light can be very specific and cause changes to the intensity of polarized light. Thus, polarimetric imaging can derive a wealth of intrinsic information about cells and tissues, e.g., morphological, biochemical, and functional properties. Without the need for sample preparation, this approach is non-destructive, enabling real-time, in situ study of biological samples. Despite the apparent information that could be obtained from circular polarization measurements, implementation of standalone circular polarimetric microscopy in biology is very limited, mainly due to the challenging imaging procedures that involve the repeated exchange of optical components for sequential recording of different polarization states.^20^

Optical metasurfaces (MSs) are planar nanostructured interfaces and have recently attracted tremendous interest due to their unprecedented capability in the manipulation of the amplitude, phase, and polarization of light at the subwavelength scale.^21−27^ Optical MSs have revolutionized design concepts in photonics, providing a compact platform to develop unusual ultrathin optical devices with multiple functionalities.^25,28,29^ To circumvent the abovementioned challenges, we propose to develop a multifunctional microscope based on a novel ultrathin optical device with multiple functionalities, which is impossible with a conventional optical element. The MS device is integrated into a microscopy system, which can simultaneously perform edge imaging, polarimetric imaging, and conventional microscope imaging. Specifically, five images with different optical properties are obtained in the same imaging plane. Because the three different imaging mechanisms are integrated into the same imaging system, this does not increase the volume of the microscope due to the compactness and multifunctionality of the optical MS device, which are realized based on both polarization and spatial multiplexing methods. We experimentally demonstrate the capability of multifunctional microscopy with various samples. Edge imaging enables fast and reliable detection of a cell. Polarimetric imaging can obtain the detailed polarization information, which can be used to resolve the microstructure (e.g., chromatin and nuclei)^30^ and their anisotropic information (e.g., orientation and ordering). The information is complementary to that obtained via conventional microscopy imaging, allowing visualization of multiple facets of samples in real-time.

## Design and Methods

**Figure 1:**
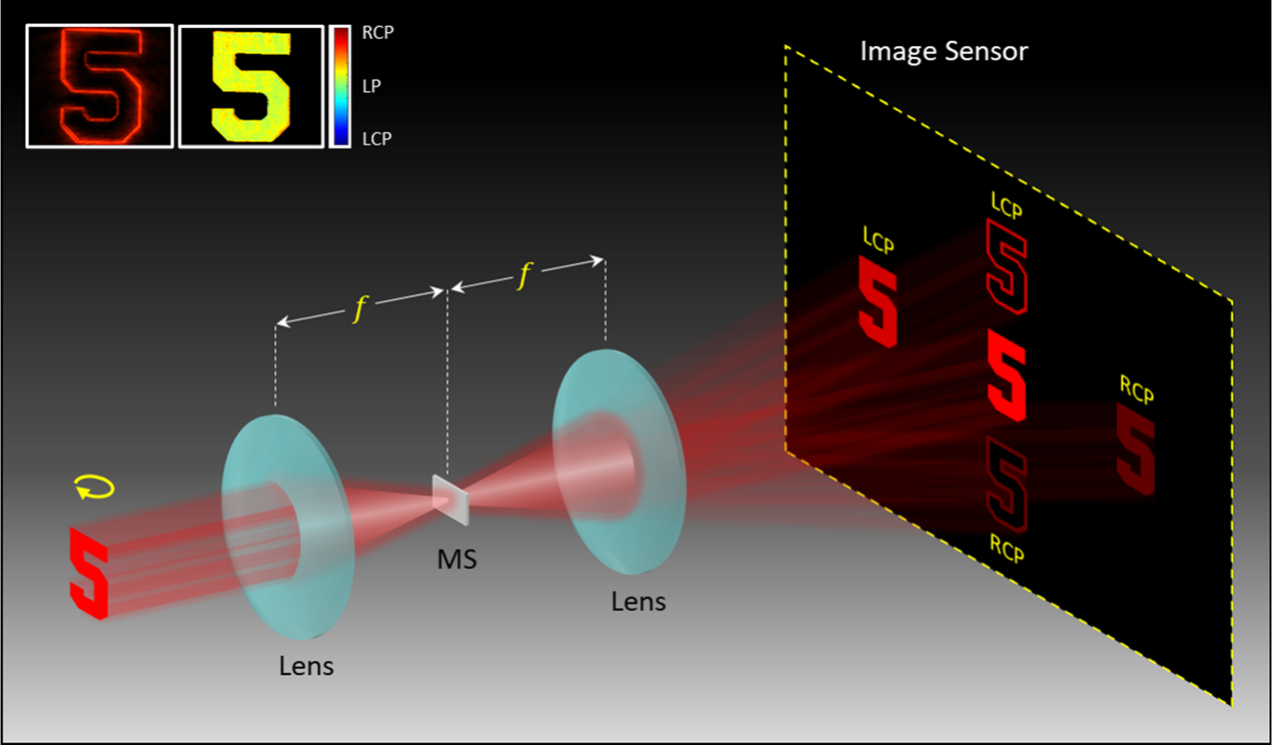
Schematic of the MS device for multifunctional microscopy. The imaging system is a Fourier transform setup, where the multifunctional MS is located in the Fourier plane. When a light beam shines on an object, five images with different optical properties are generated in the imaging plane. Along the horizontal direction, two intensity images with different circular polarizations are symmetrically distributed with respect to the normal microscope image in the middle, which arises from the non-converted part of light passing through the MS. The two intensity images with opposite circular polarizations are used to construct a polarization image, which contains spatially variant polarization information. Two edge-enhanced images with a dark background and different circular polarizations are symmetrically distributed along the vertical direction.

Figure 1 shows the concept of the multifunctional microscopy system based on a multifunctional MS device. The imaging system is a Fourier transform setup, where the multifunctional MS is placed in the Fourier plane. When a light beam shines on an object, five images with different optical properties are generated in the imaging plane. Along the horizontal direction, two intensity images with different circular polarization states are symmetrically distributed with respect to the conventional microscope image in the middle, which arises from the non-converted part of light that passes through the MS. The two intensity images with opposite circular polarizations can be used to construct a polarization image, which contains spatially variant polarization information of a scene of interest. Two edge-enhanced images with a dark background and different circular polarization states are symmetrically distributed along the vertical direction. The enhanced edge images can be used to distinguish the edges of the sample.

To realize the ultrathin multifunctional optical device, we utilize a geometric MS consisting of gold nanorods with spatially variant orientations sitting on an ITO-coated glass substrate. Each nanorod can be considered as the combination of a perfect polarizer (low percentage) and a piece of normal flat glass slab that can only add a uniform phase profile (not spatially variant phase profile).^25,31^ Thus, the Jones matrix of each nanorod (*J*_rod_) can be written as
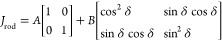
1where δ is the orientation angle of each nanorod with respect to the *x* axis.  is the unit matrix of the flat glass slab. *A* and *B* are the coefficients of the glass slab and the polarizer, respectively. When the incident light beam is circularly polarized, the output beam can be expressed as
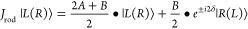
2where |*L*⟩ and |*R*⟩ represent left circularly polarized (LCP) and right circularly polarized (RCP) light, respectively. The transmitted light includes two parts: non-converted and converted parts. The non-converted part has the same polarization state as that of the incident beam, while the converted part has an additional phase shift of ±2δ generated by the geometric MS, where “+” and “–” represent the signs of the phase shift for incident LCP and RCP light, respectively. The phase shift is known as the Pancharatnam–Berry phase or the geometric phase.^32,33^ By controlling the orientation angles δ of the nanorods, the desired phase profile can be obtained.

**Figure 2:**
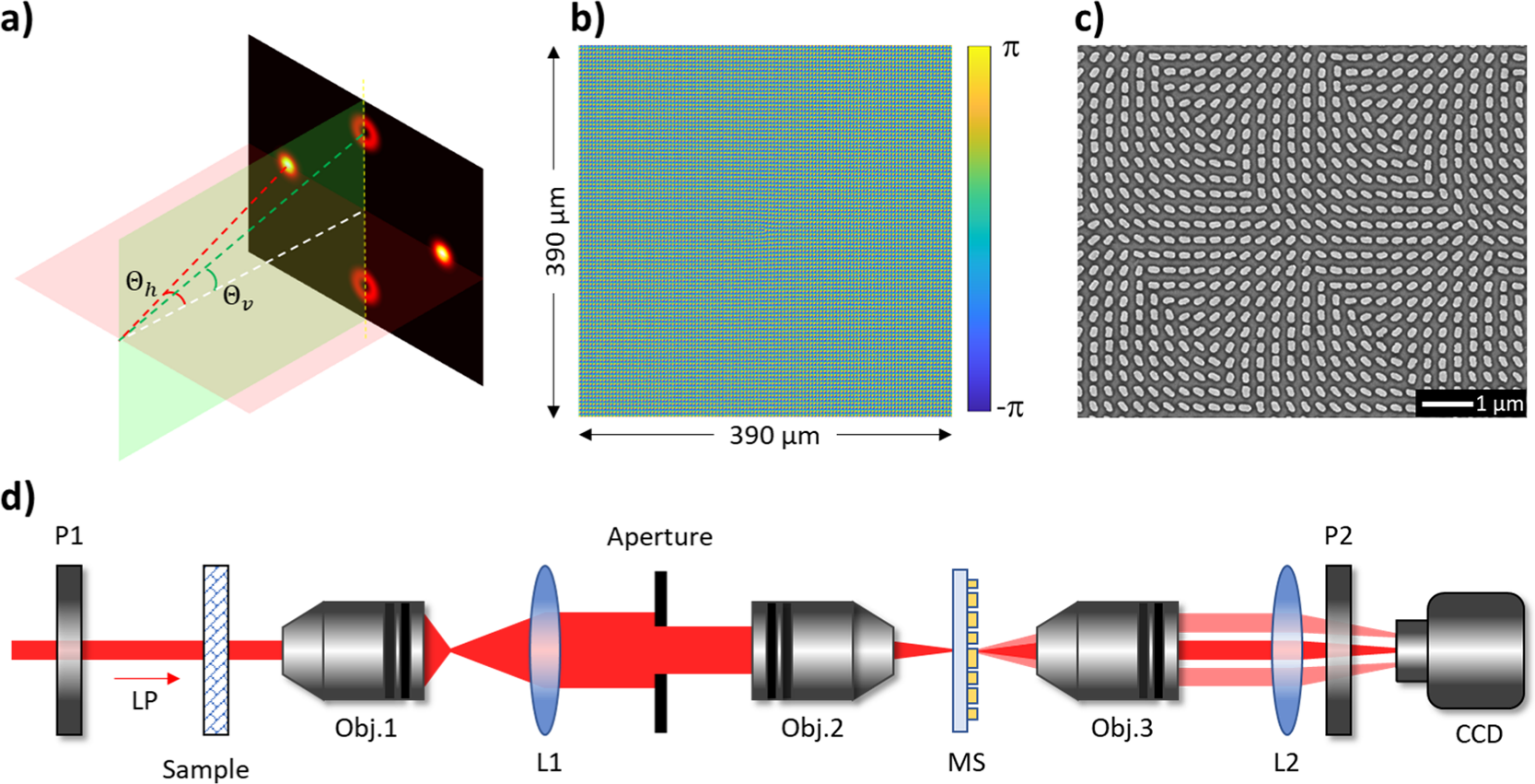
Design principle, fabricated MS, and experimental setup. (a) Geometric parameters of the phase gradient along the horizontal direction and the off-axis spiral phase profile (topological charge ) along the vertical direction. Under the illumination of LP light at 600 nm, the half off-axis angles Θ_h_ and Θ_v_ are 9.5 and 8.2°, respectively. (b) Calculated phase profile of the designed MS. (c) SEM image of the fabricated device. (d) Experimental diagram to perform a Fourier transform. P1 and P2: linear polarizers, Obj.1, Obj.2, and Obj.3: 20× objective lenses (working distance 19 mm), L1 and L2: convex lenses (*f* = 75 mm), aperture: rectangular aperture (2.5 × 2.0 mm^2^), MS: metasurface, and CCD: charge-coupled device.

The multifunctional MS is designed based on both polarization and spatial multiplexing methods. A phase gradient along the horizontal direction and a spiral phase along the vertical direction are combined together, which is realized with a geometric MS. The phase gradient can generate two images with different circular polarizations, while the spiral phase can generate two edge enhanced images with different circular polarizations along the vertical direction. Moreover, the additional phase gradient is added to the spiral phase profile to realize the off-axis design along the vertical direction. The non-converted part is along the optical axis and can generate an image with the same polarization state as that of the incident light. The phase distribution of the MS is governed by
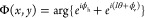
3where ϕ_h_ is the phase gradient along the horizontal direction that can generate an off-axis angle Θ_h_ with respect to the optical axis, ϕ_v_ is the phase gradient along the vertical directions that can provide the off-axis angle Θ_v_ with respect to the optical axis, θ is the azimuthal angle, and the spiral phase has a topological charge  (similar to optical vortex design). The projection geometry of the MS is shown in Figure 2a. For the incident LCP light, the multifunctional MS imprints a phase gradient (ϕ_h_) along the horizontal direction and flips the handedness of the incident polarization state to RCP. An RCP image is located on the right side. On the other hand, upon the illumination of RCP incident light, the MS imprints a phase gradient with −ϕ_h_ and again flips the handedness of the polarization to LCP, leading to an LCP image located on the left side. For linearly polarized (LP) incident light, both images appear and are symmetrically distributed with respect to the optical axis as the LP light consists of both LCP and RCP states with equal components. Similarly, two symmetrically distributed images with enhanced edges are observed along the vertical direction due to the imparted spiral phase distribution and the off-axis design . For a plasmonic MS consisting of gold nanorods, the majority on the transmission side is the light with the non-converted part, which forms the conventional microscope image in the center of the imaging plane. The multifunctional MS device is put in the Fourier plane, which enables the same imaging system to possess multiple functionalities, including polarization detection, edge enhancement, and functionality of a conventional microscope.

In the design, the phase gradient ϕ_h_ is designed to provide the off-axis angle Θ_h_ of 9.5° along the horizontal direction, which is realized with a phase difference of π/6 between neighboring pixels of the sample along the horizontal direction at 600 nm. For the edge enhancement, the topological charge  and the angle Θ_v_ are 1 and 8.2°, respectively. The value for the angle Θ_v_ corresponds to the phase difference π/7 between neighboring pixels of the sample along the vertical direction, which can generate the required phase gradient ϕ_v_. The phase profile of the designed MS can be calculated based on eq 3, which is shown in Figure 2b.

Since a geometric MS can produce a local abrupt phase change Φ = ±2δ, the orientation angles δ(*x*,*y*) of the nanorods are defined by Φ(*x*,*y*)/2. Each nanorod is 220 nm long, 130 nm wide, and 40 nm thick. The size of each pixel is 300 nm along both *x* and y directions. The designed MSs can operate in the whole visible region^31,34^ and their response spectrum is provided in the Supporting Information Section 1. The designed devices are fabricated based on the standard electron beam lithography, followed by the film deposition and the lift-off process. The fabrication details are provided in the Experimental Details. The dimension of the fabricated sample is 390 × 390 μm^2^. Figure 2c shows the scanning electron microscopy (SEM) image of the fabricated MS for the multifunctional microscope.

The diagram of an experimental setup to characterize the fabricated MS is shown in Figure 2d. A light beam with tuneable wavelengths is generated by a supercontinuum laser source (NKT Photonics SuperK EXTREME). An LP laser beam is generated using a linear polarizer (P1). A first objective (Obj.1) with a magnification of 20× and a first convex lens (L1) are used to generate an image of a sample on a rectangular aperture (2.5 × 2.0 mm^2^). A second objective (Obj.2) with a magnification of 20× is used to focus the image of the sample onto a Fourier plane where the MS is located. A third 20× objective (Obj.3) and a second convex lens (L2) are used to transform the result from the Fourier plane to an imaging plane that can be captured with a charge-coupled device (CCD) camera. A second linear polarizer (P2) acts as an analyzer and is used to modulate the intensity of the conventional microscopic image in the center of the imaging plane. The transmission axis of the polarizer P1 is fixed at 0° with respect to the *x* axis to generate an LP light beam along the horizontal direction.

## Results

**Figure 3:**
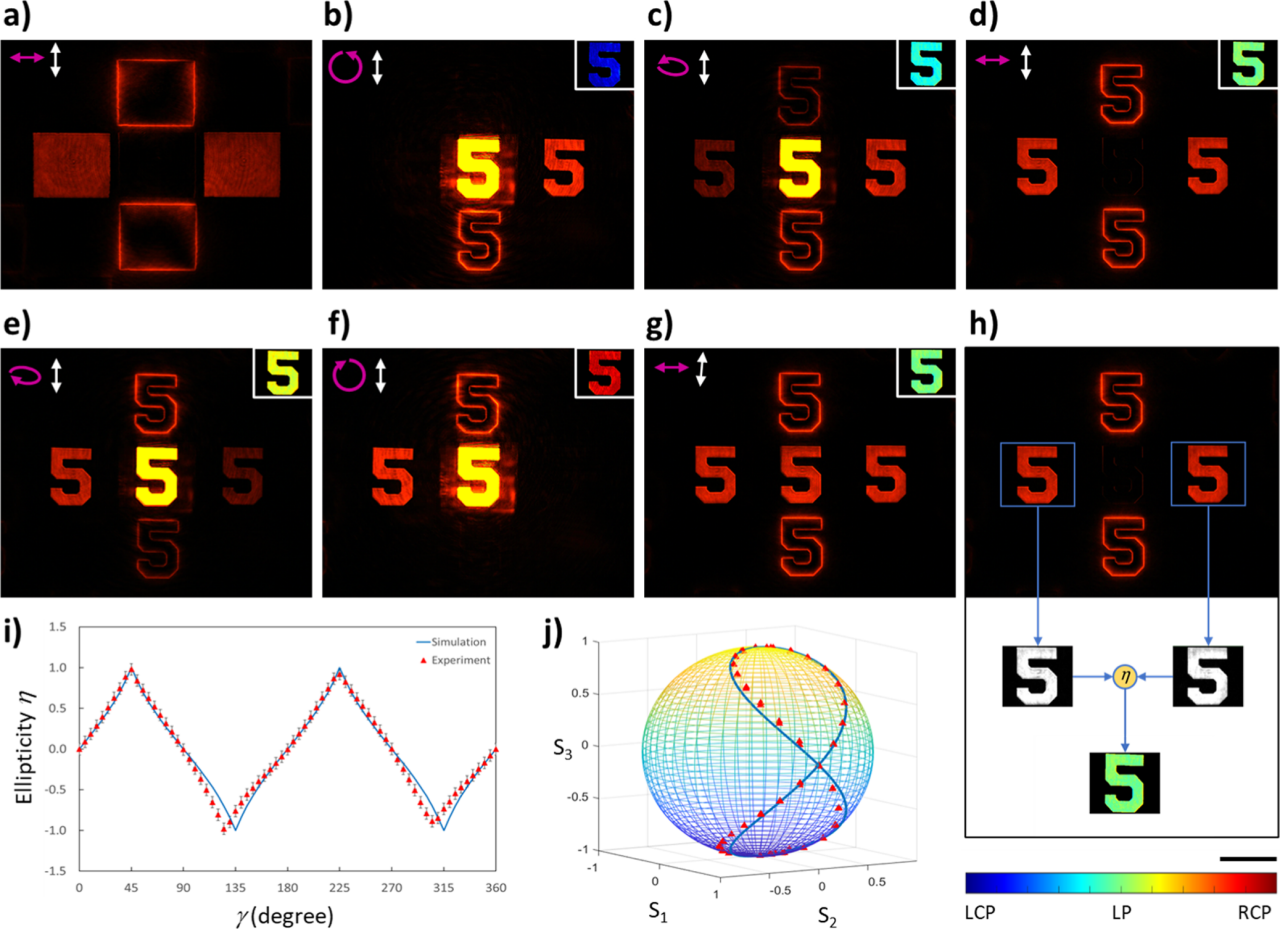
Experimental results with different polarization states of the incident light beam. (a) Captured images without a sample. Captured images of the number “5” on the negative USAF 1951 test target at different polarization states, including (b) LCP, (c) LEP, (d) LP, (e) REP, and (f) RCP. The transmission axis of the analyzer is along the horizontal direction. (g) The angle between the transmission axis of the analyzer and the *x* axis is 85°. The purple arrows and white double arrows represent the polarization states of the incident light beam and the transmission axes of the analyzer, respectively. The image intensities rise and fall according to the polarization state of the incident light since a completely polarized light beam can be decomposed into LCP and RCP components. (h) Captured images on the left and right sides are selected and converted to grayscale format for calculating the ellipticity η. A black scale bar is 300 μm. A color bar is used to represent the calculated ellipticity η shown in the inset of figure (b–g). (i) Experimentally measured ellipticities η of the number “5” vs γ (the angle between the transmission axis of a polarizer and the fast axis of a QWP). The solid curve and discreet triangles represent the simulation and experimental results, respectively. (j) A Poincaré sphere is used to show the experimentally measured polarization states (red triangles) of the incident light and theoretical data (blue solid curve).

Figure 3 shows the experimental results obtained with our multifunctional microscope. If the system is investigated without a sample, the developed MS can split the LP incident light into LCP and RCP light beams along the horizontal direction (Figure 3a). The RCP image on the right-hand side is converted from the incident LCP light, which can be used to represent the spatial distribution of the LCP component of the sample under inspection. On the other hand, the spatial distribution of the RCP component of the sample is represented on the left-hand side in Figure 3a. There are two edge images of the aperture along the vertical direction, representing the edge for the RCP component of the sample and the below one for the LCP component. Due to the LP incidence, the middle image can be removed using the analyzer with a transmission axis along the vertical direction. The rectangular aperture provides an operating area of 375 × 300 μm^2^. In our experiment, a negative USAF 1951 resolution test chart is used to characterize the functionality of the proposed optical microscope. At first, the line pairs of the group 4 element 1 on the test chart are captured as a reference position for calculating the polarization information. The details of the image alignment can be found in the Supporting Information Section 2.

In order to dynamically change the polarization state of the incident light, an additional quarter waveplate (QWP) is inserted between P1 and a sample (Figure 2d). The details can be found in the Supporting Information Section 3. The number “5” on the test target (206 μm wide and 295 μm high) is used as a sample to investigate the polarization response under different polarization states of an incident beam. The intensities of the number “5” at different positions rise and fall according to the ellipticity of various incident polarized light (Figure 3b–f), which is realized by rotating the fast axis of the QWP while the transmission axis of the polarizer is fixed. Initially, only the polarization image on the right and the edge at the bottom of the center point can be observed upon illumination of the pure LCP (Figure 3b). Gradually, we can find the polarization images on both sides along the horizontal direction and the edge images on the top and at the bottom when the sample is illuminated by the left-handed elliptically polarized (LEP) light (Figure 3c). The image intensities on the right and at the bottom are higher than those on the left and on the top. The image intensities of both sides along the horizontal and vertical directions are the same upon illumination of the LP light since it contains an LCP light beam and an RCP light beam with equal components (Figure 3d). The image intensities on the left and the top dominate when the incident light is right-handed elliptically polarized (REP) (Figure 3e). Finally, only the images on the left and the top appear when RCP light is incident on the sample (Figure 3f). Furthermore, the intensity of the image in the middle can be controlled based on Malus’ law (Figure 3g) for the incident LP beam, and the edge of the number “5” is enhanced and clearly observed. The simulations versus experiments can be found in the Supporting Information Section 4. The Fourier diffraction theory is used to evaluate the far-field intensity distributions.^35,36^

**Figure 4:**
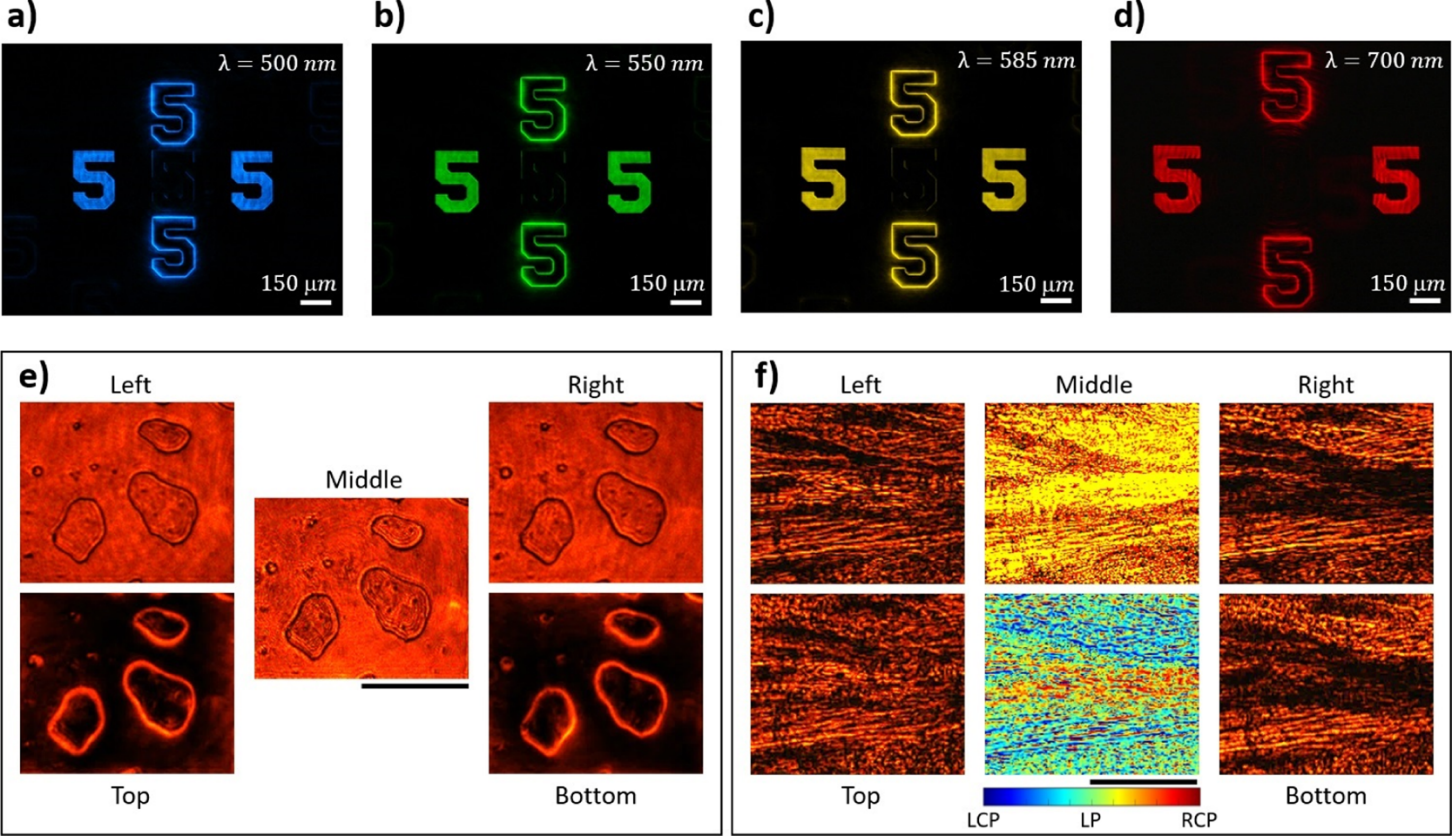
Resolution analysis, broadband performance, and imaging performance of the multifunctional microscope. Images obtained at the wavelengths of (a) 500, (b) 550, (c) 585, and (d) 700 nm. The angle between the transmission axis of the analyzer and the *x*-axis is 90°. (e) Polarization and edge images of cheek cells at the wavelength of 600 nm from the five positions of the captured image. Experimental results for the beef tendon at 600 nm are shown in (f), including the images from the five positions of the captured image, and the calculated ellipticity η. Scale bars are 150 μm.

Since the intensities of the two deflected images of the number “5” along the horizontal direction are closely related to the polarization states, both the helicity and ellipticity of those polarization states can be spatially determined by measuring the light intensities from those two CP images. As shown in Figure 3h, the captured images of the number “5” on the left and right sides are selected and converted to a grayscale format for calculating the ellipticity η. The ellipticity η and helicity of the incident light can be calculated by the intensity ratio , . η = ±1 and η = 0 correspond to RCP (LCP) and LP, respectively.^37,38^ The ellipticities versus the incident polarization (a function of γ) are obtained, where γ is the angle of the fast axis of the QWP. The transmission axis of the P1 is fixed at 0° with respect to the *x* axis. The calculated ellipticity η based on the experimental results in Figure 3b–f are shown in the inset at the top right corner. The measured and simulated results of the ellipticity η within the area of the number “5” for different angles γ are given in Figure 3i. Furthermore, the simulated and measured polarization states of the incident light are given on a Poincaré sphere (Figure 3j), which shows good agreement between the simulation and the experiment. The polarization and edge images at other wavelengths ranging from 500 to 700 nm are shown in Figure 4a–d. The change of the image quality in Figure 4d is mainly due to the imperfection of the imaging system and the increase of the designed off-axis angle with the increase of the incident wavelength.

An optical system is used to investigate the biological samples. The first sample consists of cheek cells, which are obtained by swabbing and then putting on a microscope glass slide. Five images (two LCP images, two RCP images, and one conventional microscope image) are shown in Figure 4e. Although the polarization state of light does not change much after the light passes through the cheek cells, the edges of the cells are significantly enhanced. The second sample is beef tendon, which is the piece of connective tissue that holds muscle to bone. A thin slice of beef tendon on a microscope glass slide is prepared. The experimental results are shown in Figure 4f, which clearly shows the difference between LCP and RCP images. The difference in the LCP and RCP images is mainly due to the scattering of chiral objects,^39^ which has different responses to LCP and RCP light. The calculated ellipticity (ranging from −1 to 1) in the image is given in the middle of the second row images in Figure 4f, which shows that the tendon sample is very sensitive to the light’s polarization due to the orientation of the collagen fibers.

**Figure 5:**
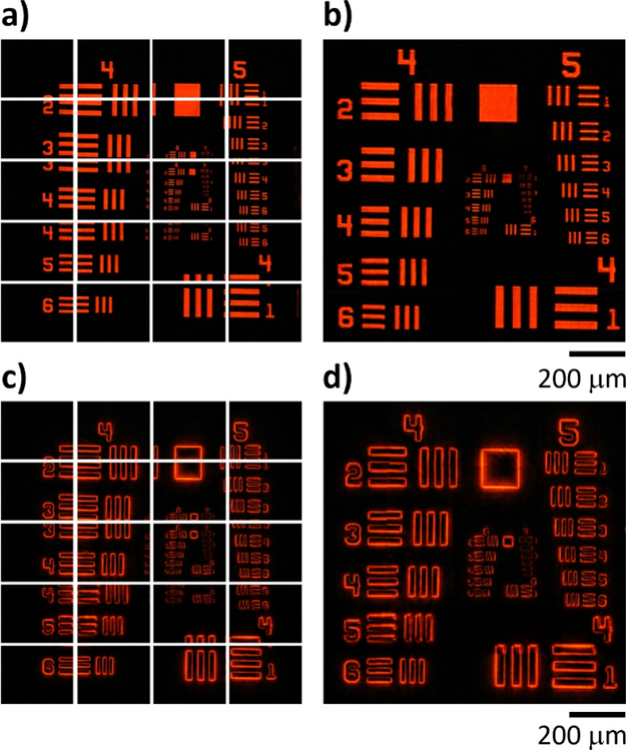
Large field size imaging with a scanning system-aided multifunctional microscope. (a) Original LCP image on the left of the imaging plane and (b) large LCP image of the resolution target based on the stitching of multiple images. (c) Original LCP edge image and (d) large LCP edge image of the resolution target. Scale bars are 200 μm.

In our experiment, the field size is 375 × 300 μm^2^. To investigate large samples, we integrate a *xy* scanning system with a sample holder in order to acquire multiple images of samples on the *xy* plane by moving the samples with a motorized stage along both horizontal and vertical directions. First, the whole image of the test target is acquired with a step size of 310 μm along the horizontal direction and 250 μm along the vertical direction. There are 20 images in total (Figure 5a). The area of an individual image is 310 × 250 μm^2^ before stitching. The large image of the sample after stitching is shown in Figure 5b. Similarly, the LCP edge images (Figure 5c) are also stitched together to generate a large image as shown in Figure 5d. Our system with a scanning approach can remarkably increase the field size of view. The large-area images of the biological samples can be found in the Supporting Information Section 5.

## Discussion

We have experimentally demonstrated a multifunctional MS device that can enable biologists to visualize the multiple facets of samples in real-time. To verify the design, we use a MS with gold nanorods with a low conversion efficiency, which can be dramatically increased by using dielectric MSs.^40,41^ It is worth mentioning that a MS with a very high conversion efficiency is not needed since the non-converted part also plays an important role in the conventional microscope image. The resolution of the imaging system is mainly determined by the numerical aperture of the objectives, the pixel size of the imaging detector, and the aperture size (Figure 2d). The involvement of the additional aperture leads to the decrease of the effective numerical aperture, which decreases the image resolution. The effect of aperture size on the image quality is provided in the Supporting Information Section 6. The effect of the amplitude contrast on the edge imaging quality is provided in the Supporting Information Section 7. Moreover, the edge imaging results with higher topological charges are provided in the Supporting Information Section 8. It is noticed that the image quality with higher topological charges becomes worse in comparison with . The imaging performance can be increased by considering the amplitude information in eq 3 in the MS design. However, the nanofabrication of such a design is quite challenging due to the involvement of nanostructures with distinct feature sizes. As a proof-of-concept demonstration, the working area we use is 375 × 300 μm^2^, which corresponds to the image dimension of 220 × 180 pixels. Due to the limited size of the MS, we use an aperture to remove the light components with high spatial frequencies, leading to the low resolution of the images, which can be further improved with a large-area MS. For the concept demonstration, the smallest feature size of a specimen that can be resolved by the system is around 15 μm. Detailed information is provided in the Supporting Information Section 9. Because the size of the collagen fibers in the beef tendon is smaller than the smallest feature size that can be resolved by the system, the edge enhancement is not observed here. The quality of large-area images can be improved by using a high-precision translational stage, a smaller scanning step (less than 310 μm along *x* and 250 μm along *y*), and an image stitching algorithm or a commercial image processing software.

One major advantage of this technique is that it can readily detect samples with a small refractive index difference from the background environment such as biological cells. Our work can bring cutting-edge technology in MSs and advanced microscopic technology to develop a compact microscopy system that can simultaneously realize edge imaging, polarimetric imaging, and conventional microscope imaging, which offer a new powerful tool for biomedical imaging, diagnosis, and inspection. The technology has a big impact in many research fields, including biomedical microscopy and crystal dislocation detection. We use single-sized nanostructures that can significantly simplify the MS design and facilitate the device fabrication process. Furthermore, MSs with various designs can be fabricated on the same glass substrate, facilitating experimental measurement by simply selecting the required design. Unprecedented information and new discoveries are expected, which will attract broad interest from the biology and life science communities. It is promising to make the technology compatible with common practice in a conventional biology lab and easy to use and thus facilitate its uptake by biologists and life scientists.

## Conclusions

To the best of our knowledge, this is the first demonstration of a 3-in-1 microscope with three different functionalities. Edge imaging can enable fast and reliable detection and recognition of a cell. Circular polarimetric imaging can obtain the detailed polarization information, which can be used to analyze the correlation between polarization and sample treatment effect. Conventional microscope imaging can monitor the real-time response of a sample to stimuli. The volume of the microscope does not increase due to the compactness and multifunctionality of the optical MS device. The system can allow users to visualize the multiple facets of samples in a single shot without liquid-crystal retarders or spatial light modulators. With its promising capabilities and potential for expandability, this microscope may open a new window for biomedical research.

## Experimental Details

The plasmonic MSs consist of gold nanorods with spatially variant orientations sitting on an ITO-coated glass substrate. First, the substrate is cleaned with acetone for 10 min and isopropyl alcohol (IPA) for 10 min in an ultrasonic bath. Then, the substrate is rinsed in deionized water and dried with a nitrogen gun. The positive poly methyl methacrylate (PMMA) 950 A2 resist is spin-coated on the substrate at 1000 rpm for 60 s followed by 1500 rpm for 15 s, producing a 100 nm-thick PMMA. After that, the sample is baked on a hotplate at 180 °C for 5 min. Electron-beam lithography (Raith PIONEER, 30 KV) is used to define nanopatterns in the PMMA film. The sample is developed in MIBK/IPA (1:3) for 45 s followed by the stopper (IPA) for 45 s. A gold film (40 nm) is deposited on the sample using an electron beam evaporator. Finally, the MSs (see Figure 2c) are fabricated after the lift-off process in acetone.

## Supporting Information Available

Conversion efficiency of transmissive MSs, image alignment, generation of incident light with various polarization states, simulations and experiments, large-area images of biological samples, the effect of aperture size on the image quality, effect of amplitude contrast on the edge detection, effect of different topological charges on the edge imaging, and resolution of the imaging system (PDF)
