# Supplementary material for: Metasurface-Enabled 3-in-1 Microscopy
Source: ACS Photonics. 2023 Jan 26;10(2):544–51. doi: 10.1021/acsphotonics.2c01971 (PMC9936625; doi:10.1021/acsphotonics.2c01971)
Supplement: Supplementary file 1 — ph2c01971_si_001.pdf [file ph2c01971_si_001.pdf]

Supporting information for

Metasurface enabled 3-in-1 microscopy

Yuttana Intaravanne[†,‡], *Muhammad Afnan Ansari*[†], *Hammad Ahmed*[†], *Narina Bileckaja*[#],

Huabing Yin[#,Φ], *and Xianzhong Chen*[†,*]

[†]Institute of Photonics and Quantum Sciences, School of Engineering and Physical Sciences, Heriot-Watt University, Edinburgh, EH14 4AS, UK

[‡]National Electronics and Computer Technology Center, National Science and Technology Development Agency, 112 Thailand Science Park, Phahonyothin Road, Khlong Nueng, Khlong Luang, Pathum Thani 12120, Thailand

[#]Biomedical Engineering Division, James Watt School of Engineering, University of Glasgow, Glasgow, G12 8QQ, UK

Supplementary Section 1. Conversion efficiency of transmissive metasurfaces

The plasmonic metasurface consists of gold nanorods with spatially variant orientations sitting on a glass substrate [1-3]. The fabricated nanorods are 220 nm long, 130 nm wide, and 40 nm high. The simulation results are obtained by using the frequency domain solver of the Computer Simulation Technology (CST) Microwave Studio software. The refraction index of the glass substrate is 1.46. The unit cell boundary is used along the x and y directions, and an open boundary is used along the z direction. The simulated and experimental results are provided in **Figure S1**. The difference between simulation and experiment is mainly due to the imperfection of the fabricated samples.

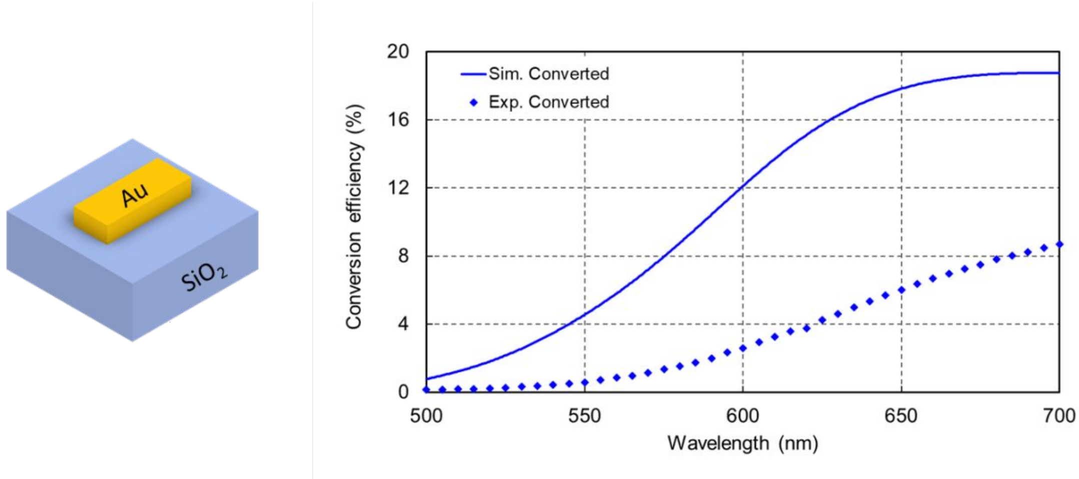

Figure S1. The designed unit cell and its simulated and measured conversion efficiencies.

Supplementary Section 2. Image alignment

At first, the line pairs of the group 4 element 1 on the negative USAF 1951 test chart are captured as a reference position. The image is then converted to a grayscale image followed by a thresholding method to create a binary image. The binary image on the left (in a red rectangle) is cropped and filled with red (**Figure S2(i)**). On the right in a green rectangle, the image is then cropped and filled with green (**Figure S2(ii)**). These red and green images are used to align the

positions of the LCP and RCP images to be overlapped while calculating the polarization information. The results in **Figure S2(iii)** illustrate the misalignment, here the positions of the red and green rectangles for image selection need to be adjusted. By shifting the position of either the red or green rectangles, a good alignment can be obtained (**Figure S2(iv)**). The positions of red and green rectangles are then kept for cropping other captured images to calculate the polarization information.

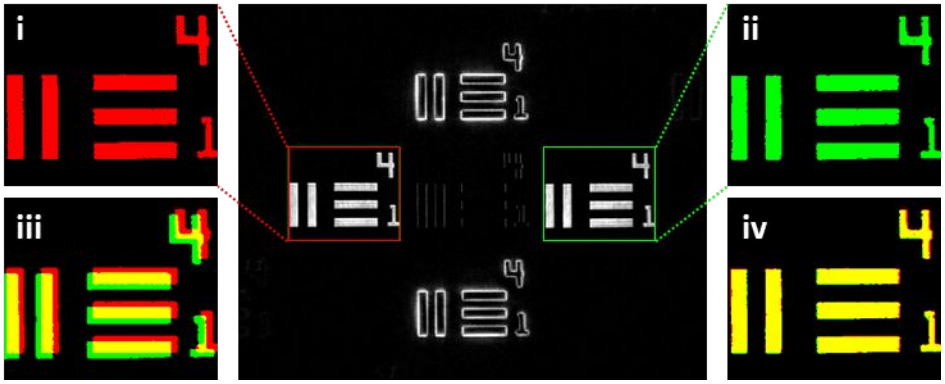

Figure S2. The captured image of group 4 element 1 on the test target for image alignment. (i) Selected area on the left side, (ii) selected area on the right side, (iii) misalignment of the images in (i) and (ii), and (iv) good alignment.

Supplementary Section 3. Generation of incident light with various polarization states

Suppose an LP light beam with a Jones vector $\begin{bmatrix} \cos\alpha \\ \sin\alpha \end{bmatrix}$ is generated when the incident light passes through the linear polarizer P1, where α is the angle between the polarization direction and the x-axis. The Jones matrix of a quarter-wave plate (J_{QWP}) is given by

$$J_{QWP} = e^{-\frac{i\pi}{4}} \begin{bmatrix} \cos^2\beta + i\sin^2\beta & (1-i)\sin\beta\cos\beta \\ (1-i)\sin\beta\cos\beta & \sin^2\beta + i\cos^2\beta \end{bmatrix} \quad \text{(S1)}$$

Where the QWP has a fast axis at angle β with respect to the horizontal axis. If we take the Jones matrix of the QWP from Equation S1 into account, the polarization state of output light after the LP light passes through the QWP can be written as

$$J_{QWP}LP = e^{-\frac{i\pi}{4}} \begin{bmatrix} \cos^2\beta + i\sin^2\beta & (1-i)\sin\beta\cos\beta \\ (1-i)\sin\beta\cos\beta & \sin^2\beta + i\cos^2\beta \end{bmatrix} \begin{bmatrix} \cos\alpha \\ \sin\alpha \end{bmatrix} \quad \text{(S2)}$$

Here, the output product can be expressed as

$$J_{QWP}LP = a\begin{bmatrix} 1 \\ -i \end{bmatrix} + b\begin{bmatrix} 1 \\ i \end{bmatrix} \quad \text{(S3)}$$

where

$$a = \frac{\sqrt{2}}{4}(\sin 2\beta\cos\alpha + \cos\alpha - \cos 2\beta\sin\alpha + i(-\cos 2\beta\cos\alpha - \sin 2\beta\sin\alpha + \sin\alpha))$$

and

$$b = \frac{\sqrt{2}}{4}(-\sin 2\beta\cos\alpha + \cos\alpha + \cos 2\beta\sin\alpha + i(-\cos 2\beta\cos\alpha - \sin 2\beta\sin\alpha - \sin\alpha))$$

are the components of RCP and LCP light, respectively. Here, different polarization states of incident light are obtained by controlling the angles α and β.

Supplementary Section 4. Simulations and experiments

The proposed imaging system is a Fourier transform setup, where the designed metasurface is put in a Fourier plane and can perform a convolution operation ($\otimes$) for an input image ($E_{in}(x,y)$). Here, an output image can be written as [4]

$$E_{out}(x,y) = E_{in}(x,y) \otimes m(r,\theta) \quad \text{(S4)}$$

where x and y are spatial coordinates of the input image. $r = \sqrt{x^2 + y^2}$ and θ are polar coordinates in the Fourier plane. $m(r,\theta)$ is the point-spread function (PSF), which can determine the filtering function of the optical system.

$$m(r,\theta) = -\frac{\pi R}{2r}[J_1(\tau)H_0(\tau) - J_0(\tau)H_1(\tau)]e^{i\theta} \quad \text{(S5)}$$

Where J_0 and J_1 are Bessel functions of zero and first order, respectively. H_0 and H_1 are Struve functions of zero and first order, respectively. R is the radius of a circular aperture, $\tau = kRr/f$, and $k = 2\pi/\lambda$. Thus, the output image can be written as

$$E_{out}(x,y) = -E_{in}(x,y) \otimes \frac{\pi R}{2r}[J_1(\tau)H_0(\tau) - J_0(\tau)H_1(\tau)]e^{i\theta} \quad \text{(S6)}$$

In the simulation, we use R = 1 mm, f = 19 mm, and $\lambda =$ 600 nm. The image in the middle and the polarization images on the left and right sides are calculated with a fixed value of θ. For edge enhancement, θ is defined as $arctan(y/x)$. The intensity of the polarization and edge images are calculated based on the components a and b in Eq. S3, while the middle image is calculated based

on the Jones vector of incident LP light and the Jones matrices of a quarter-wave plate and an analyzer as follows

$$E_{out\_middle} = e^{-\frac{i\pi}{4}} \begin{bmatrix} \cos^2\vartheta & \sin\vartheta\cos\vartheta \\ \sin\vartheta\cos\vartheta & \sin^2\vartheta \end{bmatrix} \begin{bmatrix} \cos^2\beta + i\sin^2\beta & (1-i)\sin\beta\cos\beta \\ (1-i)\sin\beta\cos\beta & \sin^2\beta + i\cos^2\beta \end{bmatrix} \begin{bmatrix} \cos\alpha \\ \sin\alpha \end{bmatrix}$$

(S7)

where ϑ is the angle between the transmission axis of the analyzer and horizontal axis. The simulation and experimental results are shown in **Figure S3**. The simulations are performed using the Fourier diffraction theory [3, 5].

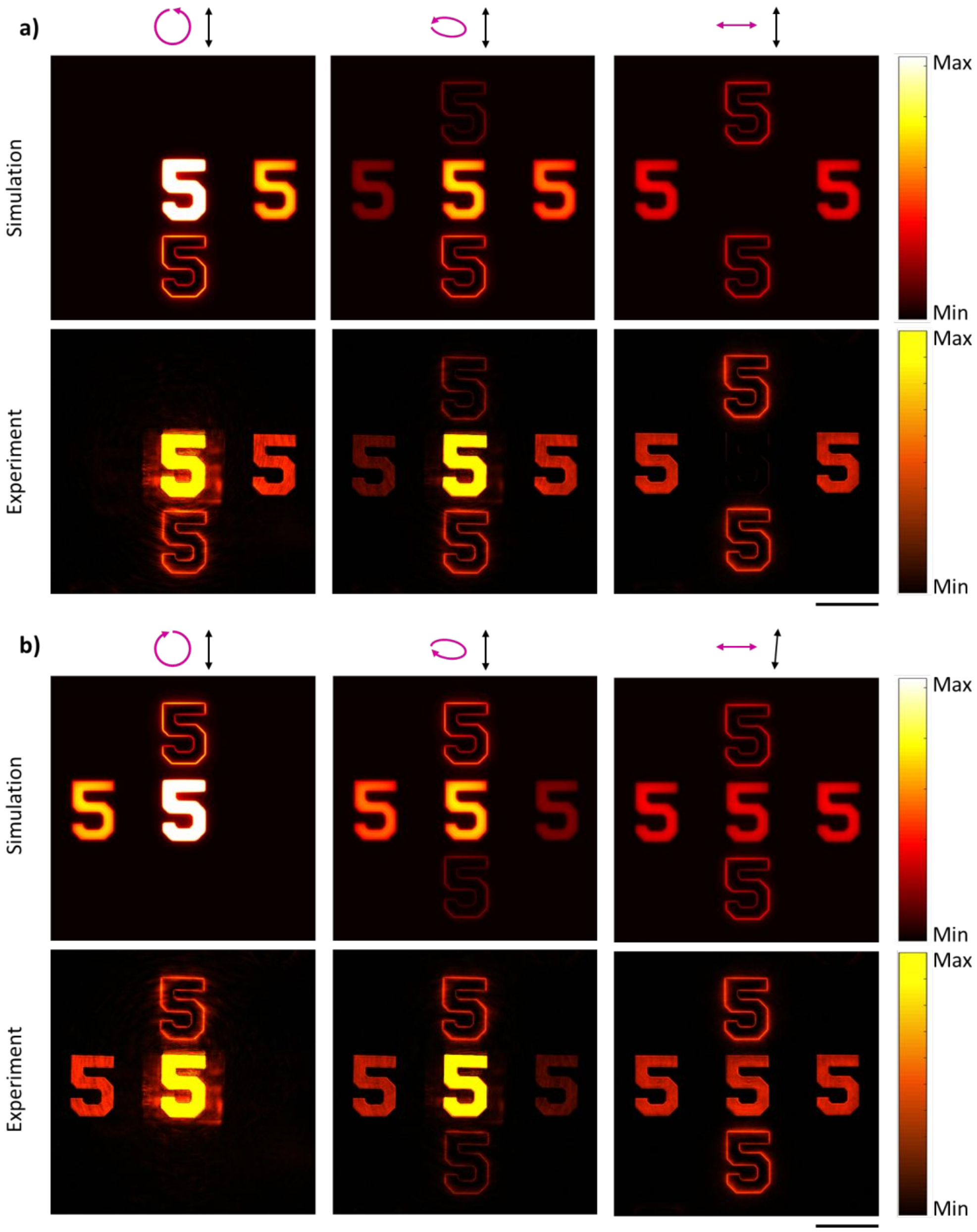

Figure S3. Simulation and experimental results of the number "5" upon the illumination of incident light with different polarization states. Pink and black arrows are the polarization states of the incident light and the transmission axis of the analyzer, respectively. Scale bars are 300 µm.

Supplementary Section 5. Large-area images of biological samples

Figure S4a and **Figure S4b** show the large LCP image and LCP edge image of cheek cells, which are obtained with 6×5 images. The polarization and edge images are created based on the multiple LCP images on the left and those on the top, respectively. The calculated ellipticity η is shown in **Figure S4c**. Experimental results for the beef tendon are given in **Figures S4d-S4f**.

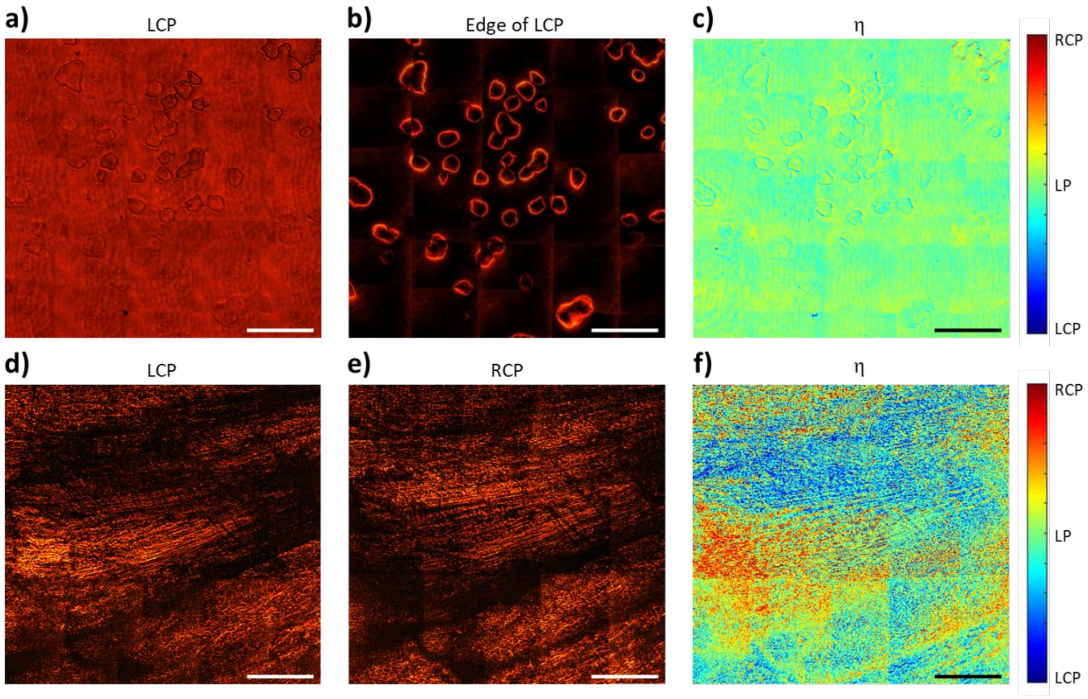

Figure S4. Large-area images of (a)-(c) cheek cells and (d)-(f) beef tendon. Scale bars are 300 μm.

Supplementary Section 6. The effect of aperture size on the image quality

As shown in Eq. S5-S6, the radius of the aperture (R) is one of the key parameters in the point-spread function that influences the quality of the output image. The image quality becomes worse with the decrease of aperture size as shown in **Figure S5**.

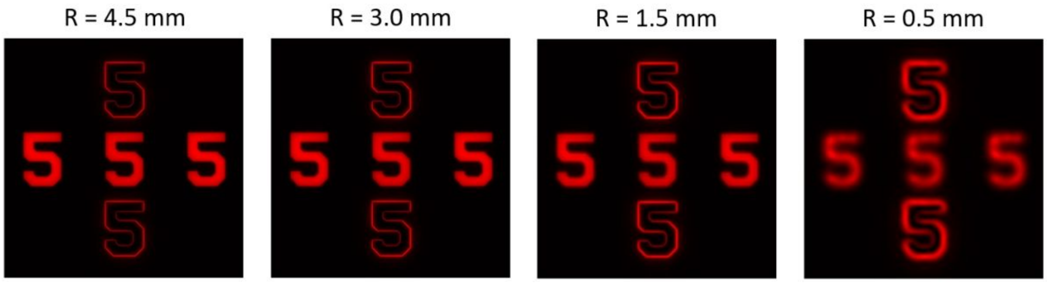

Figure S5. Simulation results with different aperture sizes.

Supplementary Section 7. Effect of amplitude contrast on the edge detection

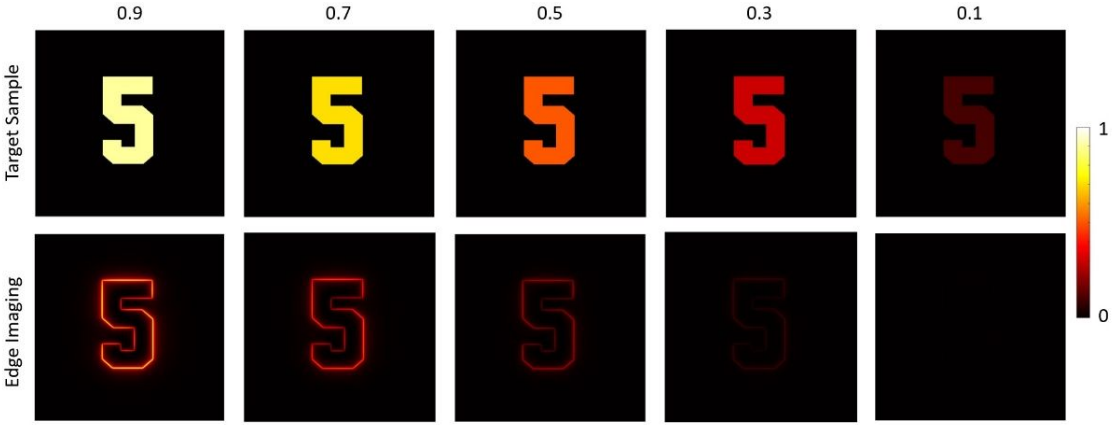

Figure S6. Effect of different values of amplitude contrast on the edge detection.

Supplementary Section 8. Effect of different topological charges on the edge imaging

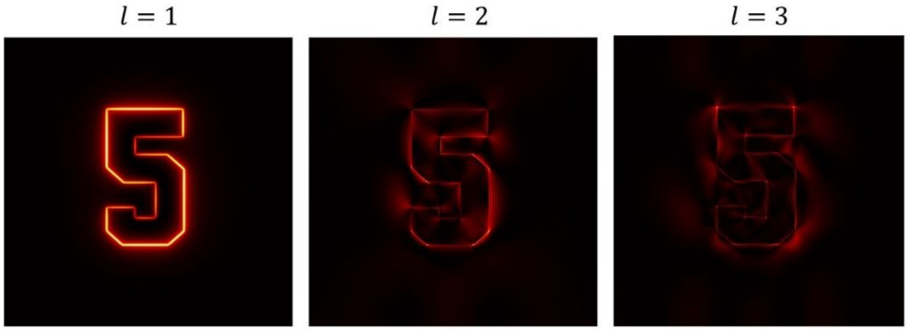

Figure S7. Results with topological charges $l = 1, 2$ and 3.

Supplementary Section 9. Resolution of the imaging system

By obtaining the line profiles across the line pairs from the polarization and edge images of the test target on the left and the top. We notice that the smallest line pairs that our optical system can be observed are in group 6 element 1 of the test target with a signal-to-noise ratio of more than 20% (**Figure S8**). This indicates that the smallest size of a specimen that can be resolved is about 15.6 μm.

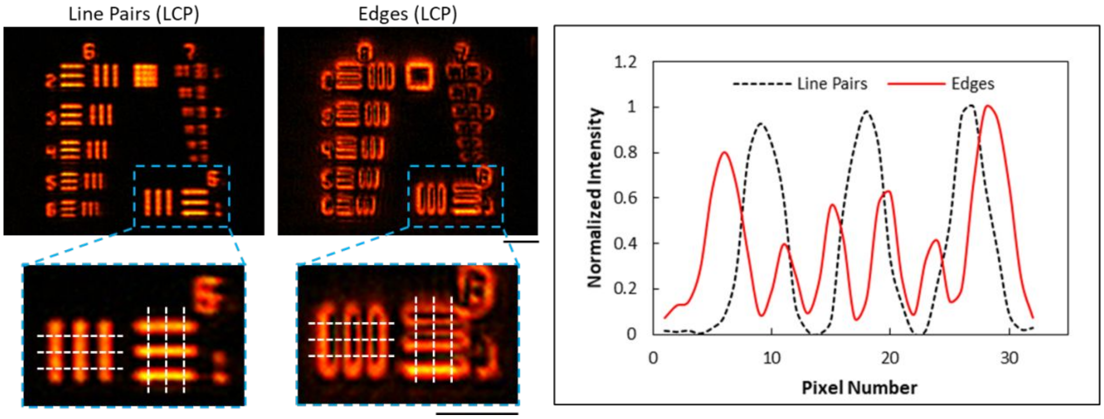

Figure S8. System resolution analysis. Scale bars are 50 μm.

REFERENCES

1. Wang, R.; Intaravanne, Y.; Li, S.; Han, J.; Chen, S.; Liu, J.; Zhang, S.; Li, L.; Chen, X., Metalens for Generating a Customized Vectorial Focal Curve. *Nano Lett* **2021,** *21* (5), 2081-2087.

2. Intaravanne, Y.; Han, J.; Wang, R.; Ma, A.; Li, S.; Chen, S.; Chen, X., Phase Manipulation-Based Polarization Profile Realization and Hybrid Holograms Using Geometric Metasurface. *Adv Photonics Res* **2021,** *2* (7), 2000046.

3. Ming, Y.; Intaravanne, Y.; Ahmed, H.; Kenney, M.; Lu, Y.-q.; Chen, X., Creating Composite Vortex Beams with a Single Geometric Metasurface. *Adv Mater* **2022,** *34* (18), 2109714.

4. Huo, P.; Zhang, C.; Zhu, W.; Liu, M.; Zhang, S.; Zhang, S.; Chen, L.; Lezec, H. J.; Agrawal, A.; Lu, Y.; Xu, T., Photonic Spin-Multiplexing Metasurface for Switchable Spiral Phase Contrast Imaging. *Nano Lett* **2020,** *20* (4), 2791-2798.
5. Jiang, Q.; Jin, G.; Cao, L., When metasurface meets hologram: principle and advances. *Adv. Opt. Photon.* **2019,** *11* (3), 518-576.
